# Amplification, Decoherence, and the Acquisition of Information by Spin Environments

**DOI:** 10.1038/srep25277

**Published:** 2016-05-19

**Authors:** Michael Zwolak, C. Jess Riedel, Wojciech H. Zurek

**Affiliations:** 1 Department of Physics, Oregon State University, Corvallis, OR 97331, USA; 2 Perimeter Institute for Theoretical Physics, Waterloo, Ontario N2L 2Y5, Canada; 3 IBM Watson Research Center, Yorktown Heights, NY 10598, USA; 4 Theoretical Division, MS-B213, Los Alamos National Laboratory, Los Alamos, NM 87545, USA

## Abstract

Quantum Darwinism recognizes the role of the environment as a communication channel: Decoherence can selectively amplify information about the pointer states of a system of interest (preventing access to complementary information about their superpositions) and can make records of this information accessible to many observers. This redundancy explains the emergence of objective, classical reality in our quantum Universe. Here, we demonstrate that the amplification of information in realistic spin environments can be quantified by the quantum Chernoff information, which characterizes the distinguishability of partial records in individual environment subsystems. We show that, except for a set of initial states of measure zero, the environment always acquires redundant information. Moreover, the Chernoff information captures the rich behavior of amplification in both finite and infinite spin environments, from quadratic growth of the redundancy to oscillatory behavior. These results will considerably simplify experimental testing of quantum Darwinism, e.g., using nitrogen vacancies in diamond.

Quantum Darwinism is a framework that allows one to understand the emergence of the objective, classical world from within an ultimately quantum Universe^1^,^2^. Objects decohere in the presence of their environment^3^,^4^,^5^, resulting in a mixture of pointer states. During this process, the environment acts as an amplification channel^6^, acquiring and transmitting redundant information about the pointer states. This occurs via the imprinting of the object’s pointer observable^7^ 
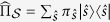
 onto conditional states, 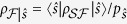, of fragments of the environment *∊*, where 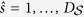 label the pointer states, 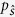 are their probabilities, and 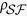 is the joint state of the system 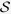 and some fragment 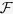.

The quantum mutual information


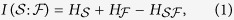


where 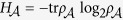 is the von Neumann entropy, quantifies the correlations generated between the system and the fragment. As was recently shown, it divides into classical and quantum components^8^, with the former being the Holevo quantity^9^,^10^ and the latter the quantum discord^11^,^12^,^13^. The Holevo quantity^9^,


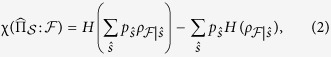


bounds the amount of classical information transmittable by a quantum channel. In our case, the classical information is about the pointer states of the system and the environment fragment’s state is the output of a quantum channel. Redundant records are available when many fragments of 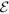 contain information about 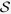, i.e., when


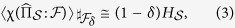


with 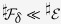 the number of subsystems in the fragment 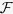 of 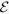 needed to acquire 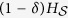 bits of information. Here, 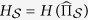 is the missing information about 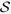, *δ* is the information deficit (the information the observers are prepared to forgo), and 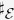 is the total number of subsystems in the environment. The average 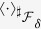 is taken over all choices of F with size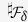.

The redundancy – the number of records of the information – is just


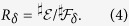


Redundancy allows many observers to independently access information about a system^14^,^15^ and guarantees that they will arrive at compatible conclusions about its state. The presence of redundancy distinguishes the preferred quantum states (that can aspire to classicality) from the overwhelming majority of possible states in Hilbert space^16^,^17^,^18^ and explains the emergence of objective classical reality in a quantum universe.

We note that, in the context of our everyday experience, 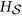 above is not the thermodynamic entropy of 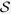. Rather, it is usually the missing information about the *relevant* degrees of freedom of 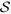. This is an important distinction: The thermodynamic entropy of a cat, for instance, will vastly exceed the information the observer is most interested in – the one bit of greatest interest in the setting imagined by Schrödinger^19^. Moreover, only such salient features of macrostates will usually be preserved or will evolve in a predictable manner under the combined influence of the self-Hamiltonian and of the decohering environment. The condition for the preservation of macrostates under copying (or under monitoring by, e.g., the environment – the cause of decoherence) is the orthogonality of the subspaces that support such macrostates^20^. It is reminiscent of the condition for preservation of microstates under measurements^21^, but the degeneracy within macrostates allows for evolution and even for the change of their thermodynamic entropy (which would certainly occur in the example of the cat we have just invoked).

To illustrate quantum Darwinism, we examine a decohering qubit. In this case, the Hilbert space of the qubit is of course too small to allow for the above distinction, leaving no room for the thermodynamic entropy. We take the interaction between the central qubit and the environment to be the pure decoherence Hamiltonian


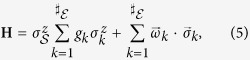


where the system’s self-Hamiltonian is assumed to be negligible and *k* specifies an environment spin. The pointer observable, 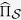, is 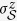. The self-Hamiltonian of the environment could be due to a magnetic field, with *ω*_*k*_ the characteristic frequency of the spin in that field. We will take 
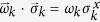
 for all specific expressions. The initial state is taken to be


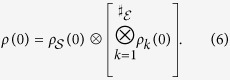


For part of this work, we will assume an environment with symmetric, time-independent couplings and initial states (*g*_*k*_ = *g*, *ω*_*k*_ = *ω*, *ρ*_*k*_(0) = *ρ*_*r*_ for all *k*). All expressions will be generalized to non-symmetric states and non-symmetric (potentially, time-dependent) couplings by taking a suitable average. In addition to giving examples of environments that are non-i.i.d. (not independent and identically distributed) and the possibility of visualizing the acquisition of partial records, this class of spin environments is the natural stepping-stone to finite, but higher dimensional, models where the information transferred into the environment pertains to coarse-grained observables of the system. Moreover, due to the prevalence of experimentally characterizable spin systems, the models discussed here will help test the underlying ideas of quantum Darwinism in a laboratory setting using, for instance, nitrogen vacancy (NV) centers^22^,^23^,^24^.

Starting from the initial product state, the system and the environment will become correlated as they interact. Observers wanting to determine the pointer state of the system, a 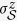 eigenstate in this case, need to distinguish the messages contained in the intercepted fragment of 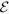. Evolution of the state, Eq. (6), generated by the Hamiltonian in Eq. (5) will result in the conditional states


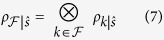


for 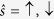. The connection between the distinguishability of these states and the Holevo quantity can be made via Fano’s inequality^10^,^25^,


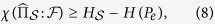


where *P*_*e*_ is the probability of error – of incorrectly identifying the conditional state. In the i.i.d. case, the quantum Chernoff bound (QCB) shows that the error probability in discriminating the two “sources” – here, the pointer states – decays as


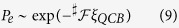


in the asymptotic regime^26^,^27^,^28^. The exponent


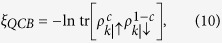


the error decay rate, is the “quantum Chernoff information” (the value of *c* is that which maximizes the exponent and satisfies 0 ≤ *c* ≤ 1). This quantity gives a generalized measure of overlap between two states.

In quantum Darwinism, the QCB provides the measure of distinguishability, including the case of non-i.i.d. environment components^6^. Amplification of information about the pointer states is reflected in the rapid decay of ignorance with the size of the fragment. Enforcing the condition, Eq. (3), together with Fano’s inequality, Eq. (8), gives an estimate for the redundancy^6^


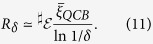


This estimate stems from a lower bound on the redundancy as *δ* → 0. The quantity 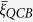 is the “typical” quantum Chernoff information (for potentially non-i.i.d. environments),


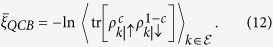


It characterizes the distinguishability averaged over contributions of *individual* environment subsystems (i.e., fragments of size 1). Equation (12) can be maximized over 0 ≤ *c* ≤ 1. This may not always be practical. We shall see it can be done for spin environments. (Previously, we demonstrated it can be done for photon environments^6^). Otherwise, though, the parameter *c* can just be set to some value between 0 and 1, e.g., *c* = 1/2, to get a weaker lower bound on the redundancy (and the quantum Chernoff information). As seen from Eqs (11) and (12), the distinguishability (alternatively, the overlap) of the conditional states, *ρ*_*k*|↑_ and *ρ*_*k*|↓_, of the *k*^th^ environment subsystem determines its contribution to the QCB and the redundancy.

Equation (11) is an extraordinarily practical tool that we will exploit in this work: To calculate the redundancy (and, hence, the amplification), one need only study *individual* environment subsystems, rather than states in the exponentially large Hilbert space of the fragment. Moreover, *the system’s probabilities appear only as small corrections to* Eq. (11) (except for the trivial case when *p*_↑_ is zero or one). Thus, the quantity of interest is the typical quantum Chernoff information 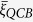, which quantifies the distinguishability of the states of the environment. *This shifts the focus from the objective existence of the state of the system to the redundancy* – *hence, accessibility by many observers, the hallmark of objectivity* – *of the records of its state in the environment. The formation and redundancy of these records depend on how the environment responds to the presence of the system*.

The quantum Chernoff information allows one to arrive at useful estimates of redundancy based on measurements of only single subsystems of the environment rather than tomography of whole fragments 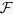, a task that is exponentially difficult in 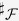. Although mathematical models of decoherence sometimes make strong assumptions about the form of the interaction Hamiltonian, the key prediction of these models – large redundancies – can be measured experimentally *without* such assumptions. Therefore, it is hoped that the results presented here will enable testing of quantum Darwinism in the laboratory.

## Results

Here we set the stage by using the quantum Chernoff information to investigate the transfer of information between a qubit system and spins of the environment. Specifically, we obtain general formulas and then use them to analyze paradigmatic examples of spin environments. We also elucidate the relation between the QCB, redundancy, and decoherence.

### Decoherence and Information

Focusing on an individual spin from the environment, we now study the relation between decoherence and the imprinting of a partial record. According to Eq. (5), the interaction with the system imprints its 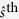 pointer state on the *k*^th^ subsystem, 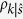, by a controlled unitary, i.e., rotating it from its initial state to the state


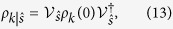


with


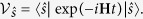


**Figure 1:**
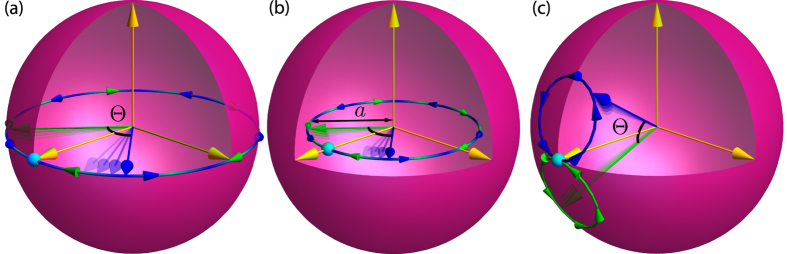
Record acquisition by an environment spin: Three-dimensional trajectories on the Bloch sphere that depict the acquisition of a record by a single two-level (spin) environment subsystem. (**a**) A qubit system interacts with a spin environment subsystem with *ω*_*k*_ = 0. (See Eq. (5).) The two conditional states of an individual environment spin, *ρ*_*k*|↑_ (green) and *ρ*_*k*|↓_ (blue), rotate in opposite directions from the initial state (light blue sphere) due to interaction with the spin-up and spin-down pointer states of the system. For pure states, the decoherence and completeness of the record is determined by the angle Θ between the Bloch vectors for *ρ*_*k*|↑_ and for *ρ*_*k*|↓_ – the angle that appears in the quantum Chernoff bound (QCB), Eq. (19). (**b**) The same as (**a**), but with an initially mixed subsystem state. The mixedness contracts the Bloch vectors (here, to a length *a* = 11/16) and reduces the ability of an environment spin to store distinguishable records of the system’s pointer states. (**c**) Same as (**a**) but with a subsystem self-Hamiltonian, 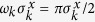. The latter contribution to the Hamiltonian can enhance or reduce the susceptibility of the subsystem to be rotated by 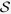, depending on the initial state, time, etc. While the case of *ω*_*k*_ = 0 gives analogous behavior to photons, the case of *ω*_*k*_ ≠ 0 is relevant for more general environments, such as the nuclear spin environment of a nitrogen vacancy (NV) in diamond.

This process of controlled rotation is depicted in Fig. 1. The off-diagonal elements of 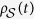 will be suppressed by the decoherence factor, 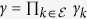, with contributions


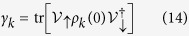


from each subsystem *k*. Each contribution to the QCB is, ignoring the logarithm,


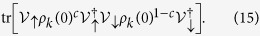


For a pure initial state (or a purified state where the observer has access to the purifying system), we thus have


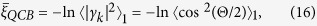


where the second equality is for spin environments and the average 〈·〉_1_ is over the individual components of the environment, i.e., fragments of size 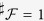. The angle Θ is how much the conditional states of the environment spin are separated on the Bloch sphere, as shown in Fig. 1. The redundancy is therefore


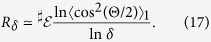


There is thus a direct correspondence between decoherence and redundancy when the environment is initially pure – when there is decoherence, records of the system’s pointer states will be proliferated into the environment. The estimate of *R*_*δ*_ using Eq. (11) comes from a lower bound on the redundancy. However, in the case of an initially pure 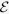 state, Eq. (11), and hence Eq. (17), is exact as *δ* → 0. This is easily shown by expansion of the mutual information, as shown in the Methods. Any initial mixedness in the relevant subspace of the environment subsystem – in the space that acquires the record – will decrease the information about 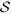 observers can deduce, as we will now show.

### The Quantum Chernoff Information for Mixed Environments

To find the QCB and *R*_*δ*_ for an initially mixed spin environment, we examine 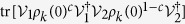, which appears in Eq. (12). Letting 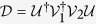 with 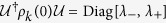, this quantity is given by


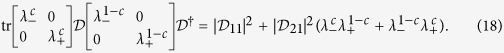


This is symmetric about *c* = 1/2 and attains its minimum there (and thus it maximizes Eq. (12)). For higher dimensional environment subsystems the minimum will not necessarily occur at *c* = 1/2, even for pure decoherence. Further, without loss of generality, let *ρ*_*k*_(0) be along the *z*-axis of the Bloch sphere and 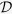 be a rotation about an axis in the *xy*-plane. One then obtains


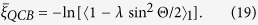


The quantity


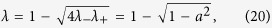


where *a* is the length of the Bloch vector, is a measure of the mixedness of the state. Unless otherwise stated, the quantities *λ*, Θ, etc., depend on the environment subsystem *k*.

*No External Field, ω*_*k*_ = 0: For *ω*_*k*_ = 0 in the Hamiltonian (5), the QCB takes on the form


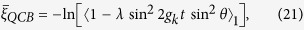


where *θ* is the angle of the initial environment spin state from the *z*-axis. Notice that the polar angle, *ϕ*, does not appear in 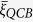 when no external field is present, and hence the QCB is rotationally symmetric about the *z*-axis. This axis is special: It is insensitive to the state of the system, as the *z*-states are eigenstates of the Hamiltonian and these states have zero capacity to acquire information (in some sense, they are the pointer states of the *environment spin*^7^).

**Figure 2:**
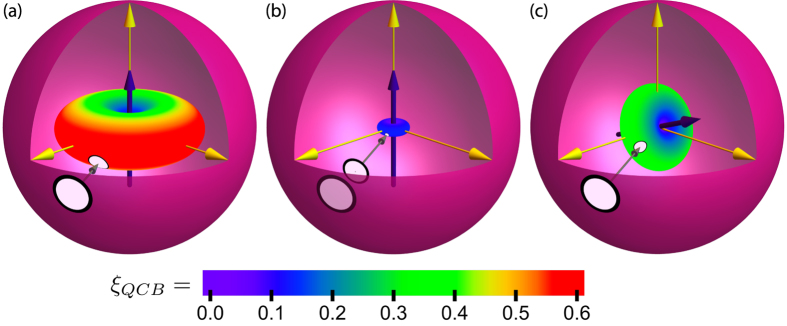
The contribution to the quantum Chernoff information, Eqs (12) and (19), for a single environment spin *k* with (**a**) *ω*_*k*_ = 0 & *a* = 1, (**b**) *ω*_*k*_ = 0 & *a* = 11/16, and (**c**) *ω*_*k*_ = *π*/2 & *a* = 1. (In all cases, *t* = 15*π*/64 and *g*_*k*_ = 1/2). These parameters are the same as in Fig. 1. The white patches map a region of initial states of the spin, specified by (*a*, *θ*, *ϕ*) in the Bloch sphere, to a region (*ξ*_*QCB*_, *θ*, *ϕ*) of the central, toroidal structure. For the mixed state case, two patches are shown: (1, *θ*, *ϕ*) in light pink and (*a*, *θ*, *ϕ*) in white. These structures demonstrate that there is only a single axis – an “insensitive axis” shown as a dark purple arrow – of initial states that have no information transferred to them, and, consequently, do not contribute to the redundancy. When *ω*_*k*_ = 0, this axis is the *z*-axis – these states of the environment subsystem cannot decohere the system and have zero susceptibility to acquire information. In a sense, they are the pointer states of the environment subsystem with respect to decoherence induced by the system. For *ω*_*k*_ ≠ 0, the insensitive axis is time-dependent due to the intrinsic dynamics of the environment. These structures show explicitly that redundancy is a universal feature of pure decoherence models; the initial states that preclude the acquisition of a partial record form a set of measure zero. In other words, essentially all spins in the environment will be imprinted with a partial record of the system’s state. (See Fig. 1 for an illustration of this process). These partial records can be therefore investigated experimentally by tomography of individual spins.

Figure 2(a,b) shows the QCB mapped from the Bloch sphere for an initially pure and mixed environment spin. The QCB forms a toroidal structure around the *z*-axis. Only along this axis is the QCB zero. In other words, all the possible initial states of the environment will proliferate redundant information, except ones that have all subsystems initially in *z*-eigenstates or mixtures thereof. Redundancy is thus inevitable, as these structures show.

*With an External Field, ω*_*k*_ ≠ 0: For 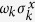 in the Hamiltonian (5), the quantum Chernoff information comes out to be


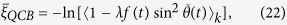


where


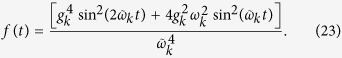


In this expression, we have used the effective field strength, 
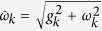
, felt by the environment spin. The factors in the average depend on the initial state and parameters describing the *k*^th^ environment subsystem. For completeness, note that 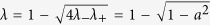 is the same factor as in the case *ω*_*k*_ = 0. The angle 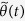 is the angle of the initial state of the environment subsystem from the “insensitive axis” at time *t*, which is defined by the Bloch angles θ* = 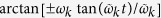 and 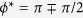 (i.e., it depends on the time and parameters of the *k*^th^ environment subsystem’s Hamiltonian). When the environment subsystem initially points in the direction of this axis, then at time *t* it will contain no information and therefore will have zero contribution to the redundancy. This (time-dependent) axis is thus the counterpart to the *z*-axis when *ω*_*k*_ = 0.

Figures 1(c) and 2(c) show the acquisition of a record and 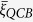 for *ω* = *π*/2, respectively. While the behavior is different – the “toroids” rotate – one still gets redundancy. The shape is also still highly symmetric. This is clear from the expression, Eq. (22), above: At any given time, the object is rotationally symmetric about the axis defined by 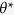 and 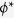. Thus, Eq. (22) shows that the Hamiltonian defines a unique structure on the Bloch sphere of individual environment spins. The spatial extent of the structure determines the redundancy achievable by states on 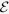, and there is always a zero point defined by the “insensitive axis”, around which the structure is also rotationally symmetric. Moreover, this demonstrates that redundancy is not fragile in the sense of being prohibited by self-Hamiltonians of the individual environment spins. Although the field *ωσ*^*x*^ can interfere with the ability of the environment to decohere the system, the structures in Fig. 2 show that fields can actually enhance the ability of some states to both decohere the system and acquire information about it. Of course, if the field is strong enough, the environment spin’s state rotates uncontrollably and will neither decohere nor acquire information about the system (this can be seen from Eq. (23), 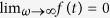, giving 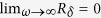). Again, this shows that only in particular cases – cases of measure zero – can redundancy vanish.

### Examples

The general results presented above set the stage for a direct application of the QCB to example spin environments. They display a variety of behaviors for the redundancy and the acquisition of records about the system’s state. Here we will discuss natural spin environments relevant to bringing quantum Darwinism into the lab.

### Gaussian Decoherence and *R*_*δ*_ ∝ *t* ^2^

Spin/two-level environments typically arise in solid state systems where a central system, such as another spin, interacts with many environment spins/two-level systems with a bounded total energy. Decoherence in this paradigmatic setting was studied in Refs. 29, 30, 31, where it was shown that this universally results in Gaussian decoherence. In this situation, the Hamiltonian is Eq. (5) and the coupling constants depend on the environment size as


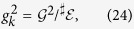


with 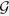 implicitly dependent on *k*. We note that the coupling constants do not actually need to depend on environment size to get Gaussian behavior. However, this dependence is physical (and also makes a short time approximation unnecessary). We will set *ω*_*k*_ = 0 for convenience. The decoherence factor of the system decays as


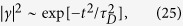


where *τ*_*D*_ is the decoherence time. This can be derived using already demonstrated results.

Taking the relation between the QCB and the decoherence factor *for pure states*, Eq. (16), we can write


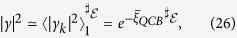


where the first approximate equality becomes exact in the limit 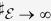 since |*γ*_*k*_|^2^ approaches 1 with corrections that can be written in a series in 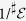. The 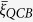 is given by Eq. (21) by letting the environment be pure (*λ* = 1),


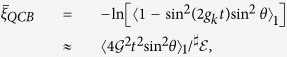


where we have used Eq. (24) and assumed that 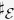 is very large. Again, all environment parameters implicitly depend on *k*. Equation (26) now becomes Eq. (25) with


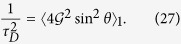


This is the same result as in Refs. 29, 30, 31 taking into account the different definitions (the coupling *g*_*k*_ in the Hamiltonian is defined in Refs. 29, 30, 31 as *g*_*k*_/2 and 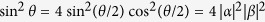, with implicit *k* dependence). Note that for a mixed state environment with the same 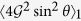, the decoherence time will be the same, i.e., the decoherence – in contrast to amplification and, hence, redundancy – is independent of the mixedness of the environment.

For the redundancy, including arbitrary mixedness, Equation (11) becomes


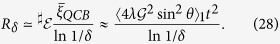


**Figure 3:**
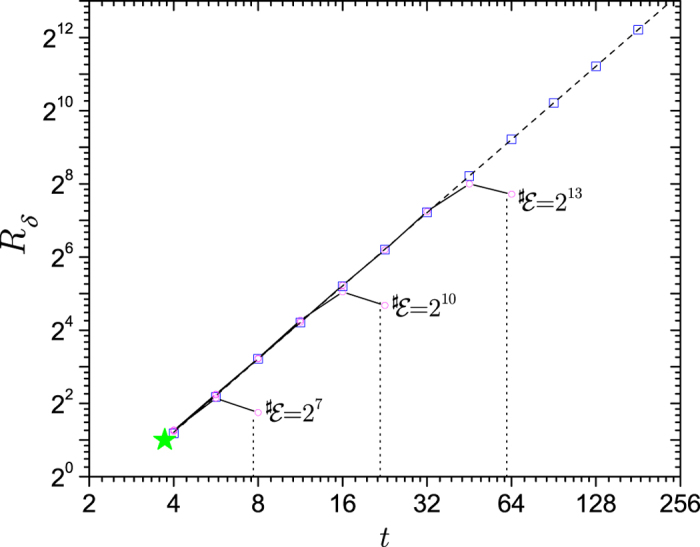
The redundancy vs. time for Gaussian decoherence with an initially pure environment. Here, the couplings 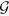 are chosen uniformly from the interval [−2, 2], which gives 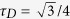. The other parameters are *p*_↑_ = 1/2 and *δ* = 10^−16^. The blue squares are from computing the Holevo quantity with very large environments and the black line is the QCB result, Eq. (29). The green star is the redundancy onset time, Eq. (30). The three black dashed lines are for different finite 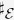 (for a fixed realization of the random coupling constants). For finite environments, there is a quadratic growth of the redundancy up to the recurrence time, 
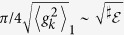
, where information flows from the environment back into the system. The recurrence time for each of the finite environments is indicated by the black dotted lines. Thus, even for finite environments, one can expect to see signatures of Gaussian behavior in the redundancy.

**Figure 4:**
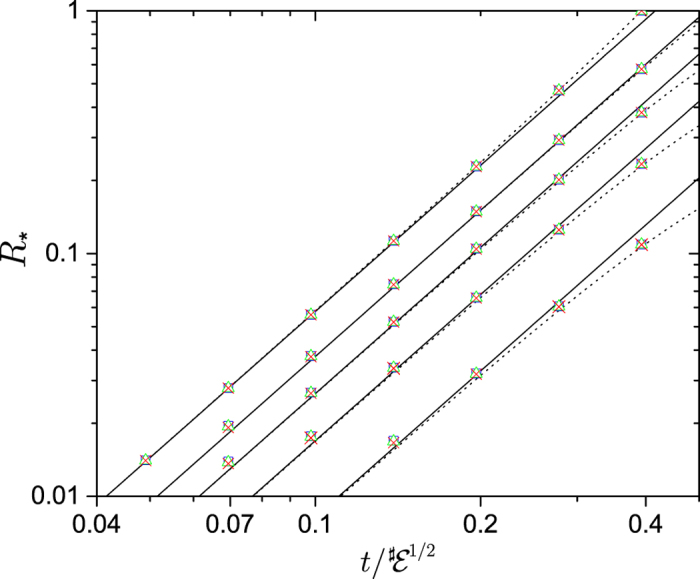
The amplification efficiency quantified by the quantum Chernoff information. Here, the amplification is taken relative to a reference case, 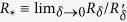. The QCB predicts that the relative efficiency is 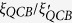 with *ξ*_*QCB*_ from Eq. (19) (black, dotted lines). The quadratic growth of the redundancy, given by Eq. (29), is shown as black, solid lines. For all data, the QCB prediction and quadratic growth match well with the numerically computed results. For very small relative efficiencies there is some deviation, which is due to the finite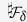 obtainable numerically. The full QCB result deviates from quadratic behavior for very long times (i.e., on the order of the recurrence time of an individual spin interaction, which goes as 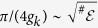). The data in the figure is as follows: The five lines are for varying haziness (the initial entropy *h* = *H*[(1 + *a*)/2] of a single environment spin^32^,^33^) *h* = 0, 1/5, 2/5, 3/5, and 4/5 from top to bottom. Each set of symbols shows the numerical result for 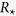 for three initially pure states of the system (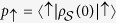 = 1/2, 1/8, and 1/32) relative to the QCB reference case with with *h* = 0 and *t* = *π*/8. Further details can be found in the Methods.

Assuming that the distributions of *λ* and the 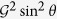 are independent,


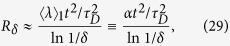


where *α* = 〈*λ*〉_1_ is a spin analog of receptivity (see below). This indicates that redundancy grows quadratically with time. Moreover, for 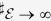, redundancy grows indefinitely and without bound, even though the interaction energy between the system spin and the infinite environment is bounded. Figure 3 shows the growth of redundancy with time for both finite and (effectively) infinite environments. Figure 4 shows the redundancy for initially mixed environments plotted with the results found using numerically exact techniques for finite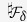. This figure shows that the exact results approach the QCB as *δ* gets smaller (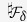 gets bigger).

In addition to the long time behavior for very large 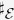, we can also determine when redundant records start to form. From Eq. (29), the onset of redundancy, 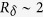, happens at


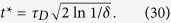


That is, it is essentially the decoherence time multiplied by a factor weakly dependent on the information deficit *δ*. The latter is order one for a large range of information deficits *δ*. Figure 3 marks this onset time with a green star.

Before we discuss another example, we note that the quadratic growth in the redundancy is in sharp contrast to the linear growth for photon environments^6^,^34^,^35^,^36^. Photons (or photon-like environments) are also amenable to calculations using the QCB^6^. In this case, the redundancy grows as


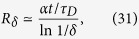


where *α* is the receptivity of the environment to making records, which is a dimensionless quantity determined by the mixedness and angular distribution of the incoming photon states^6^,^34^,^35^,^36^. The growth of the redundancy for photons is due to the linear increase of the environment size in time, with each individual environment component (each photon) acquiring a partial record of fixed fidelity, at least on average. This is in contrast to the spin environment which has a fixed size and continuously interacts with the system. The quadratic growth in redundancy is due to the increasing fidelity of the partial records with time. We will show elsewhere that a flux of spins can represent the same acquisition of information as photon environments – and thus be used as a stand-in for photons.

We note that there is a receptivity for both spins and photons. However, for spins it is simply *α* = 〈*λ*〉_1_. Other factors that affect the ability of the environment spins to acquire information also affect their ability to decohere the system. This different form of the receptivity is due to the fact that we have not made the equivalent of a “weak scattering” approximation, but rather have only made a weak coupling approximation for each environment spin and have allowed each spin to continuously interact with the system.

### Other Non-i.i.d. Environments

**Figure 5:**
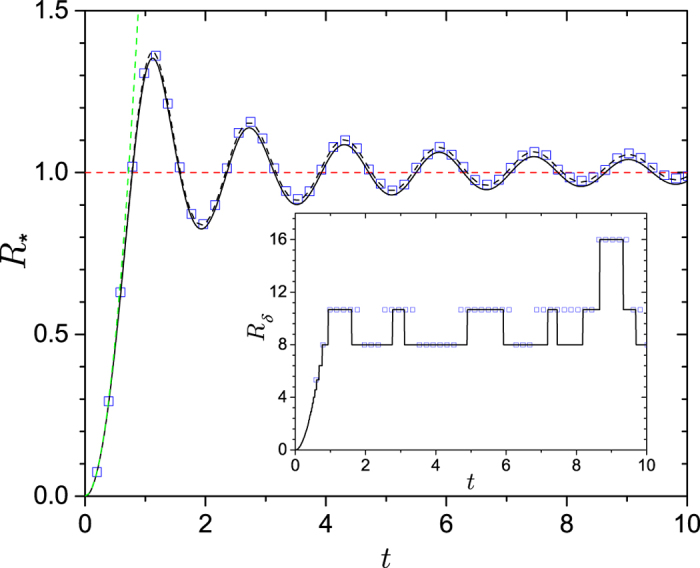
The amplification efficiency quantified by the quantum Chernoff information. As with Fig. 4, the amplification is taken relative to a reference case, 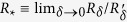. The plot shows the numerical data (blue squares), QCB (black solid line), Gaussian regime (green dashed line), and an approximation that has corrections for finite *δ* (black dashed line). The red dashed line is the *t* → ∞ result. When the coupling constants *g*_*k*_ come from a band of energies, [0, 1], the efficiency of amplification initially increases quadratically with time – i.e., it is in the universally present Gaussian regime – and then develops into an oscillatory behavior. The oscillations appear due to drawing the coupling constants from a finite band. In this case, information flowing into spins with large couplings returns to the system, i.e., there is a fixed recurrence time. The inset shows *R*_*δ*_ for 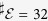 and *δ* = 10^−1^ for a *single* set of spins with random coupling constants drawn from [0, 1]. The exact numerical data, the open blue squares, shows that the oscillatory features are still present even for this small environment. Moreover, the black line shows a discretized application of the QCB. This shows that in potential experiments with a very limited number of subsystems of the environment can still display intricate dynamics of the redundancy and the emergence of objective information. Moreover, the QCB can capture this behavior and thus eliminate the need for a full tomographic characterization of the system and environment. The Methods section gives details of the data in the figure.

The Gaussian decoherence case above is not the only possible realization of a central spin continually interacting with a fixed set of spins. When a large number of environment spins couple strongly to the system, then one can have still different behavior. Consider, for instance, the Hamiltonian in Eq. (5) with *ω*_*k*_ = 0 and the coupling constants *g*_*k*_ randomly drawn from the interval [0, *W*], with *W* the “bandwidth”. The QCB result, Eq. (11), readily yields


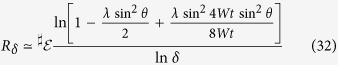


by averaging 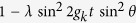 over the coupling constants assuming that *λ* and *θ* are constant. Figure 5 shows the QCB result for the redundancy compared with the results from averaging the Holevo quantity. As *δ* is decreased, the numerical results converge to the QCB result. Notice that the environment spins have a band of energies, which is responsible for both the oscillation frequency and decay.

In this setting – environment spins strongly coupled to the system – 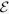 will often have only a finite number of spins, i.e., 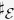 can not be taken to be infinite. The qualitative features given by the QCB, however, will still be present in the redundancy even for relatively small number of spins in the environment, as shown in the inset of Fig. 5. Therefore, one can expect that in some settings, more intricate dynamics of the redundancy will be present.

## Discussion

The QCB demonstrates that redundancy is inevitable under pure decoherence: The only way to avoid it for a spin environment is for all spins to be in a completely mixed state (i.e., *a* = 0, implying *λ* = 0) or for all spins to be precisely aligned with the insensitive axis (i.e., Θ = 0). Figure 2 shows this graphically. Pure decoherence always gives rise to the redundant proliferation of information except in rare – measure zero – cases. Furthermore, we showed that the redundancy using the QCB estimate, Eq. (11), agrees with numerically exact results, which covers a wide variety of behavior from Gaussian decoherence to oscillations. We discuss further examples in a forthcoming publication.

Although unavoidable imperfections ensure that real-world systems never perfectly satisfy pure decoherence, Eq. (5), models like the one in Ref. 18 show that redundancy emerges even in the presence of other types of environmental interactions. The results presented here will help shed light on experiments where decoherence and amplification are expected to occur for spins, such as NV-centers immersed in an environment of nuclear spins. Spin models also help in understanding the historical generalization of quantum Darwinism^37^.

While the QCB gives the exact asymptotic redundancy for the models here, two key features of the estimate, Eq. (11), are that (a) it does not rely on idealized initial states or Hamiltonians when considered as a lower bound and (b) it can be computed using only bit-by-bit measurements, with no need for complicated multipartite tomography. Thus, whether a system self-Hamiltonian is present or not, and whether there are more complicated interactions, one can demonstrate information transfer into the environment with experimentally feasible measurements. Our results, especially when confirmed experimentally, further elucidate the acquisition of information by the environment and show why perception of a classical objective reality in our quantum Universe is inescapable^6^,^38^.

## Methods

The numerical computation in Fig. 3 are for a symmetric environment with *θ* = *π*/2 and *g*_*k*_ = 1 for all *k* in order to make use of the method of Refs. 32,33. Since *g*_*k*_ = 1 for all *k*, the decoherence time is *τ*_*D*_ = 1/2, which when rescaled by 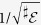 will be zero for all practical purposes (and well off the scale of the figure). We note that 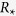 is found numerically by fitting the decay of 
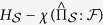
 in order to more rapidly approach the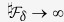 (*δ* → 0) limit. The numerical technique is exact for the computation of the Holevo quantity, 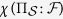, for finite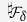. Even though the QCB is the asymptotic result (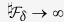), the numerical and analytical data match.

The data for Fig. 5 are calculated as follows: The reference case is evaluated at *t* → ∞. The environment is taken to be pure. The numerical data (open blue squares) was found by sampling the random distribution of spins 10^8^ times to obtain *δ* as a function of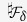. The solid black curve is the QCB, Eq. (32) and the dashed black curve is from the expansion of the Holevo quantity (see the following paragraph below). The dashed green curve is Gaussian decoherence regime, giving 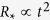. The information deficit, *δ* = 10^−10^, is still finite and thus there is a gap between the QCB and the exact results, which closes as *δ* becomes smaller. Note that the actual redundancy is quite large, linear in the environment size. For the inset, the numerical data was evaluated using an exact average of the Holevo quantity over all subsets of size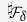 of the 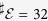 spins for a fixed realization of the random coupling constants. The discretized application of the QCB takes 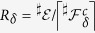, where 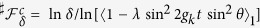 is a continuous version of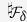, takes the ceiling (i.e., makes it discrete), and where the average is over the single realization of random coupling constants. The redundancy jumps between discrete steps since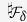 takes on integer values, e.g., *R*_*δ*_ = 16 is for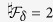 and *R*_*δ*_ = 32/3 ≈ 10 for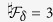, etc.

For a pure system and environment, and a decoherence process due to the Hamiltonian in Eq. (5), the mutual information is given by 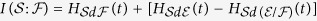, with the term in square brackets being the quantum discord^8^,^33^, and the Holevo quantity by 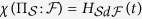. Here, the entropies are the entropy of the system only decohered by some component of the environment, either 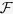, 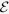, or 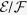. These expressions can both be generalized to the case of the system being mixed, see Eq. (A13) of Ref. 33. Expanding 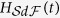, inputing it into Eq. (3), and using that 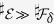 – so that each of the spins in the fragment can be treated independently in the average – gives 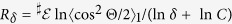 with 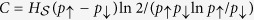. *C* → ln 4 for *p*_↑_ = *p*_↓_ = 1/2. This equation is, for any practical *δ*, exact and shows that the redundancy approaches the QCB result from above as *δ* → 0. This equation is the black dashed line in Fig. 5.

## Additional Information

**How to cite this article**: Zwolak, M. *et al*. Amplification, Decoherence, and the Acquisition of Information by Spin Environments. *Sci. Rep*. **6**, 25277; doi: 10.1038/srep25277 (2016).
